# Ultra-strong spin–orbit coupling and topological moiré engineering in twisted ZrS_2_ bilayers

**DOI:** 10.1038/s41467-022-31604-w

**Published:** 2022-08-22

**Authors:** Martin Claassen, Lede Xian, Dante M. Kennes, Angel Rubio

**Affiliations:** 1 Department of Physics and Astronomy, University of Pennsylvania, Philadelphia, PA 19104 USA; 2 Songshan Lake Materials Laboratory, 523808 Dongguan, Guangdong China; 3 Max Planck Institute for the Structure and Dynamics of Matter, Center for Free Electron Laser Science, 22761 Hamburg, Germany; 4 Institut für Theorie der Statistischen Physik, RWTH Aachen University and JARA-Fundamentals of Future Information Technology, 52056 Aachen, Germany; 5 Center for Computational Quantum Physics, Simons Foundation Flatiron Institute, New York, NY 10010 USA

**Keywords:** Quantum fluids and solids, Electronic structure, Electronic properties and materials, Topological insulators

## Abstract

We predict that twisted bilayers of 1T-ZrS_2_ realize a novel and tunable platform to engineer two-dimensional topological quantum phases dominated by strong spin-orbit interactions. At small twist angles, ZrS_2_ heterostructures give rise to an emergent and twist-controlled moiré Kagome lattice, combining geometric frustration and strong spin-orbit coupling to give rise to a moiré quantum spin Hall insulator with highly controllable and nearly-dispersionless bands. We devise a generic pseudo-spin theory for group-IV transition metal dichalcogenides that relies on the two-component character of the valence band maximum of the 1T structure at Γ, and study the emergence of a robust quantum anomalous Hall phase as well as possible fractional Chern insulating states from strong Coulomb repulsion at fractional fillings of the topological moiré Kagome bands. Our results establish group-IV transition metal dichalcogenide bilayers as a novel moiré platform to realize strongly-correlated topological phases in a twist-tunable setting.

## Abstract (web summary)

Studies of twisted bilayer transition metal dichalcogenides have so far focused only on those containing group-VI metals. Here, the authors predict that twisted bilayers of ZrS_2_, with the group-IV metal Zr, form an emergent moiré Kagome lattice with a uniquely strong spin-orbit coupling, leading to quantum-anomalous-Hall and fractional-Chern-insulating states.

## Introduction

Twisted van der Waals heterostructures have recently emerged as an intriguing and highly tunable platform to realize unconventional electronic phases in two dimensions^1–4^. Spurred by the discovery of Mott insulation and superconductivity in twisted bilayer graphene^5,6^, remarkable progress in fabrication and twist-angle control has led to observations of correlated insulating states or superconductivity in a variety of materials, including trilayer and double-bilayer graphene, homo- and hetero-bilayers of twisted transition metal dichalcogenides (TMDs)^7–18^, and heterostructures at a twist on hexagonal boron nitride substrates^19,20^. At its heart, this rich phenomenology stems from electronic interference effects due to the moiré superlattice, which can selectively quench kinetic energy scales to realize almost dispersionless bands, permitting a twist angle controlled realization of regimes dominated by strong electronic interactions. At the same time, the drastic reduction of kinetic energy of the low-energy moiré bands implies straightforward gate-tunable access to a wide range of filling fractions, permitting wide-ranging experimental access to the phase diagrams of paradigmatic models of strongly-correlated electrons^2^. Consequently, the putative realization of strongly-correlated electron physics in a tunable setting has garnered significant attention, resulting in growing experimental evidence for novel correlated phases, including unconventional superconductivity^4,21,22^.

Notably, and despite negligible intrinsic spin–orbit coupling in graphene, these were found to include topological states of matter. Here, the realization of the interaction-induced quantum anomalous Hall effect without external magnetic fields in twisted bilayer^23–25^ and trilayer^26^ graphene has spurred numerous proposals for more exotic fractionalized topological states of matter^27–30^, which however rely on a delicate interplay of spontaneous ferromagnetic order, valley polarization, and substrate engineering effects to induce the requisite nontrivial band topology. Generalizations to twisted transition-metal dichalcogenides have focused on telluride-based group VI compounds with 2H structure in the monolayer which exhibit an intrinsic quantum spin Hall effect^31^, with the quantum anomalous Hall effect recently observed^32^ and similarly expected to emerge from spontaneous valley polarization^33,34^.

Central to the present work, we demonstrate for twisted bilayer ZrS_2_ with 1T structure that the paradigm of twist-controlled suppression of the bare kinetic energy scales can be straightforwardly extended to instead promote *spin–orbit coupling* to constitute the dominant energy scale at low energies, opening up a new and exotic regime for experimental and theoretical investigation. Remarkably, we find that the two-component character of the valence band maximum in such two-dimensional group IV transition metal dichalcogenides enters in an essential manner, leading to the emergence of a clean *moiré Kagome* lattice with almost dispersionless quantum spin Hall bands at small twist angles. We demonstrate that this tunable realization of a ZrS_2_ moiré heterostructure with strong spin-orbit coupling and strong interactions can therefore provide a robust and novel platform to probe the profound interplay of non-trivial band topology and electronic correlations, and shed light on elusive quantum phases beyond the purview of conventional condensed matter systems.

## Results

### Emergent Kagome moiré pattern in twisted ZrS_2_ bilayers

**Fig. 1:**
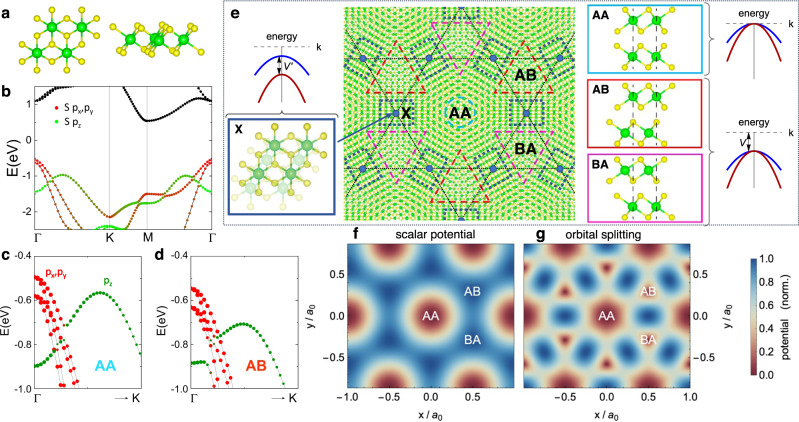
Moiré patterns of twisted ZrS_2_ bilayers. **a** Top and side view of the 1T structure of ZrS_2_. **b** The band structure of ZrS_2_ monolayer hosts a valence band maximum at Γ composed of the *p*_*x*_, *p*_*y*_ chalcogen orbital *J* = ± 3/2 states, with a spin-orbit splitting of ~ 100 meV. **c**, **d** In aligned bilayers with AA or AB stacking, the valence band maximum at Γ (composed of of antibonding states of *p*_*x*_, *p*_*y*_ symmetry) is energetically separated by ~ 50meV from bonding *p*_*x*_, *p*_*y*_ states and *p*_*z*_ orbitals. Hence, antibonding *p*_*x*_, *p*_*y*_ states compose the top-most moiré valence bands at small twist angles. **e** In small-angle twisted heterostructures of ZrS_2_, the interlayer alignment interpolates from AA to AB/BA stacking as a function of position in the moiré unit cell. The *p*_*x*_, *p*_*y*_ antibonding states remain degenerate for both AA and AB/BA stacking in the absence of spin-orbit coupling. Their overall energetic shift is encoded in **f** as an effective scalar moiré superlattice potential, with minima that form a moiré honeycomb lattice. **g** Crucially, the loss of rotational symmetry away from local AA, AB stacking further splits the *p*_*x*_, *p*_*y*_ states. This orbital splitting contribution to the moiré potential is maximal in three “domain wall” regions “X” in the moiré unit cell [(e)]; upon exceeding the scalar potential, electrons in the top-most moiré valence band form an emergent moiré Kagome lattice [dashed lines in **e**; guide to the eye].

ZrS_2_ is a group IV transition metal dichalcogenide with an exfoliable layered structure. Different from group VI TMDs such as Mo_2_ and *WS*_2_ that normally adopt a 2H layered structure, ZrS_2_ has a stable 1T structure^35^ in its ground state as shown in Fig. 1a, without distorting into the 1T’ structure^36,37^. Bulk ZrS_2_ is a semiconductor with a band gap of 1.80 eV^38,39^ and it remains semiconducting when thinned down to the monolayer^35,40^. In contrast to group-VI transition metal dichalcogenides such as MoS_2_ with 2H structure, the valence band maximum in ZrS_2_ and other group-IV transition metal dichalcogenides is located at Γ already in the monolayer and is composed of twofold degenerate chalcogen *p*_*x*_, *p*_*y*_ orbitals. Spin–orbit coupling lifts their degeneracy and introduces a ~100 meV gap [Fig. 1b]. This property readily carries over to aligned bilayers with symmetric AA and AB stacking configurations [Fig. 1c, d]; here, the valence band maximum at Γ follows from antibonding combinations of the out-of-plane chalcogen *p*_*x*_, *p*_*y*_ orbitals. These are energetically separated from bonding combinations by ~80−100 meV [Fig. 1c, d], with a secondary local valence band maximum of *p*_*z*_ orbitals furthermore located close to Γ and similarly detuned by ~50 meV for AA stacking.

In twisted bilayers, the atomic interlayer registry interpolates continuously between local AA, AB, and BA alignment as a function of position and a moiré pattern with three-fold rotation symmetry forms [Fig. 1e]. At sufficiently small twist angles, the energetic considerations for aligned bilayers discussed above immediately suggest that the top-most (highest energy) moiré valence bands should be similarly composed of antibonding *p*_*x*_, *p*_*y*_ chalcogen orbitals. If spin-orbit coupling is neglected, these are degenerate at Γ in both AA and AB regions [Fig. 1e] by virtue of rotation symmetry. However, the valence band edge differs between the two stackings, with the smooth interpolation between local alignments in the moiré unit cell encoded in an effective periodic *scalar* moiré potential *V*(**r**) [Fig. 1f]. Minima of *V*(**r**) is located at the AB and BA regions and form an effective honeycomb lattice. Notably, for the purposes of capturing the highest-energy moiré valence bands, the potential retains to an excellent approximation of the full sixfold rotation and mirror symmetries of the monolayer, even though the macroscopic crystal is chiral. This situation is in principle analogous to twisted bilayer MoS_2_^41,42^, which hosts a series of almost dispersionless bands of Mo ${d}_{{z}^{2}}$ orbital character on an emergent moiré honeycomb lattice.

Crucially, the loss of rotational symmetry away from local AA, AB stacking lifts the orbital degeneracy between *p*_*x*_, *p*_*y*_ antibonding orbitals (in the absence of spin-orbit coupling), introducing a second energy scale into the problem. In stark contrast to 2H TMD bilayers, the two-component character of the Γ valley states enters in an essential manner. From symmetry considerations, their orbital splitting is expected to be maximal in three “domain wall” regions “X” per moiré unit cell in Fig. 1e, in which the transition-metal atoms of both layers form “stripes”, with the rotational symmetry of the local stacking order reduced to C_2_. Contrary to the scalar moiré potential, maxima of orbital *p*_*x*_, *p*_*y*_ splitting hence form a Kagome pattern [Fig. 1g]. Remarkably, if the resulting energetic gain exceeds the scalar potential *V*(**r**), it becomes favorable for charge to migrate from the honeycomb AB/BA regions to “X” regions, realizing an *emergent Kagome lattice* of *s*-like moiré orbitals in a highly-tunable setting [Fig. 1e].

### Continuum model of twisted ZrS_2_

A minimal continuum model of this scenario readily follows from the above symmetry considerations as

1 $$\hat{H}={\hat{H}}_{0}+{\hat{H}}_{{{{{{{{\rm{soc}}}}}}}}}+{\hat{H}}_{{{{{{{{\rm{pot}}}}}}}}}$$

where ${\hat{H}}_{0}$ describes the two-fold degenerate antibonding *p*_*x*_, *p*_*y*_ chalcogen states

2 $${\hat{H}}_{0}=-\frac{{\hslash }^{2}}{2{m}^{\star }}\left\{({k}_{x}^{2}+{k}_{y}^{2})\hat{{{{{{{{\bf{1}}}}}}}}}+\eta \left[({k}_{x}^{2}-{k}_{y}^{2}){\hat{{{{{{{{\boldsymbol{\tau }}}}}}}}}}_{z}+2{k}_{x}{k}_{y}{\hat{{{{{{{{\boldsymbol{\tau }}}}}}}}}}_{x}\right]\right\}$$

with the orbital degree of freedom represented via Pauli matrices $\hat{{{{{{{{\boldsymbol{\tau }}}}}}}}}$. Here, *m*^⋆^ denotes the effective average band mass, and $\eta =\frac{{m}_{+}-{m}_{-}}{{m}_{+}+{m}_{-}}$ parametrizes the ratio of light (*m*_−_) and heavy (*m*_+_) hole *p* bands at Γ. Atomic spin–orbit interactions

3 $${\hat{H}}_{{{{{{{{\rm{soc}}}}}}}}}=\frac{{\lambda }_{{{{{{{{\rm{soc}}}}}}}}}}{2}\,{\hat{{{{{{{{\boldsymbol{\tau }}}}}}}}}}_{y}{\hat{{{{{{{{\boldsymbol{\sigma }}}}}}}}}}_{z}$$

lift the orbital degeneracy, opening up a gap at Γ as discussed in detail below. Here, ${\hat{{{{{{{{\boldsymbol{\sigma }}}}}}}}}}_{z}$ acts on spin. Central to the emergence of the Kagome lattice, the moiré potential acts nontrivially on the orbital pseudospin, and can generically be written as a Fourier expansion

4 $${\hat{H}}_{{{{{{{{\rm{pot}}}}}}}}}=\mathop{\sum}\limits_{n}{V}_{n}{f}_{n}^{0}({{{{{{{\bf{r}}}}}}}})+\mathop{\sum}\limits_{n}{V}_{n}^{\prime}\left[{\hat{{{{{{{{\boldsymbol{\tau }}}}}}}}}}_{x}{f}_{n}^{(x)}({{{{{{{\bf{r}}}}}}}})+{\hat{{{{{{{{\boldsymbol{\tau }}}}}}}}}}_{z}{f}_{n}^{(z)}({{{{{{{\bf{r}}}}}}}})\right]$$

Here, *n* indexes the *n*-th moiré Brillouin zone. *V*_*n*_ parameterizes the Fourier modes of the scalar potential in direct analogy to twisted WS_2_^42^, with ${f}_{n}^{(0)}({{{{{{{\bf{r}}}}}}}})=\cos ({{{{{{{{\bf{b}}}}}}}}}_{n,1}{{{{{{{\bf{r}}}}}}}})+\cos ({{{{{{{{\bf{b}}}}}}}}}_{n,2}{{{{{{{\bf{r}}}}}}}})+\cos ({{{{{{{{\bf{b}}}}}}}}}_{n,3}{{{{{{{\bf{r}}}}}}}})$ chosen to retain the full sixfold rotation symmetry and **b**_*n*,*i*_ describing the three reciprocal lattice vectors *i* = 1, 2, 3 (related via *C*_3_ rotations) to the *n*-th Brillouin zone.

The pseudospin ${\hat{{{{{{{{\boldsymbol{\tau }}}}}}}}}}_{x}$, ${\hat{{{{{{{{\boldsymbol{\tau }}}}}}}}}}_{z}$ contributions to the potential are related in the presence of (approximate) mirror symmetry, with ${f}_{n}^{(x)}({{{{{{{\bf{r}}}}}}}})=-\frac{\sqrt{3}}{2}\cos ({{{{{{{{\bf{b}}}}}}}}}_{n,1}{{{{{{{\bf{r}}}}}}}})+\frac{\sqrt{3}}{2}\cos ({{{{{{{{\bf{b}}}}}}}}}_{n,3}{{{{{{{\bf{r}}}}}}}})$ and ${f}_{n}^{(z)}({{{{{{{\bf{r}}}}}}}})=\frac{1}{2}\cos ({{{{{{{{\bf{b}}}}}}}}}_{n,1}{{{{{{{\bf{r}}}}}}}})-\cos ({{{{{{{{\bf{b}}}}}}}}}_{n,2}{{{{{{{\bf{r}}}}}}}})\;+\frac{1}{2}\cos ({{{{{{{{\bf{b}}}}}}}}}_{n,3}{{{{{{{\bf{r}}}}}}}})$. The salient physics is encoded already in the lowest harmonic—with ${{{{{{{{\bf{b}}}}}}}}}_{1,1}=[2\pi ,-2\pi /\sqrt{3}]/{a}_{0}$, ${{{{{{{{\bf{b}}}}}}}}}_{1,2}=[0,4\pi /\sqrt{3}]/{a}_{0}$, ${{{{{{{{\bf{b}}}}}}}}}_{1,3}=[2\pi ,2\pi /\sqrt{3}]/{a}_{0}$ and *a*_0_ the moiré lattice length, the scalar potential hosts two minima in the AB and BA regions at ${{{{{{{\bf{r}}}}}}}}=[1/2,\pm 1/(2\sqrt{3})]$. Conversely, the pseudospin potential that determines the splitting of *p*_*x*_, *p*_*y*_ orbitals has three maxima in the Kagome X regions at ${{{{{{{\bf{r}}}}}}}}=[1/2,0]{a}_{0},\,[1/4,\sqrt{3}/4]{a}_{0},\,[3/4,\sqrt{3}/4]{a}_{0}$. Up to an overall energy scale, a minimal continuum model that includes only the first harmonic will therefore depend on just three dimensionless parameters *η*, $2V{m}^{\star }{a}_{0}^{2}/{\hslash }^{2}$, $2{V}^{\prime}{m}^{\star }{a}_{0}^{2}/{\hslash }^{2}$, where *a*_0_ ∝ *θ*^−1^ scales with the twist angle. Local lattice relaxation effects are encoded in the higher harmonics of the potential; as the local stacking of AB and BA regions arise energetic favorable, lattice relaxation results in large domains with almost uniform AB or BA stacking [see domains highlighted with red and purple dashed lines in Fig. 1e] with the salient stacking variation in the X regions. These parameters can be obtained by fitting the band structure obtained from DFT calculations as described below.

**Fig. 2:**
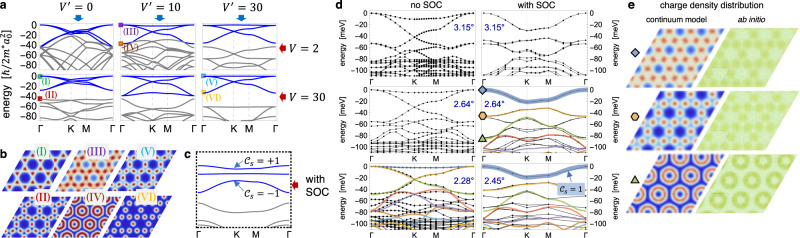
Emergent Kagome lattice and continuum theory of twisted ZrS_2_ bilayers. **a** Anatomy of the moiré band structure in the two-orbital pseudospin continuum theory, as a function of the scalar (*V*) and pseudospin (${V}^{\prime}$) potential, with *η* = 0.15 and energies normalized to $\hslash /2{m}^{\star }{a}_{0}^{2}\equiv 1$. In the absence of ${V}^{\prime}$, a well-separated honeycomb lattice with two *p* orbitals per sublattice emerges with increasing *V* [bottom left], directly analogous to twisted bilayer graphene. **b** The calculated charge density of bands (I) is localized in a honeycomb pattern of AB/BA regions; however, the next lower-energy band (II) already exhibits a Kagome charge density pattern. Conversely, the pseudospin moiré potential ${V}^{\prime}$ favors charge patterns localized in (III) the Kagome “X” regions as well as on rings (IV) around the Kagome hexagons. As *a*_0_ ~ *θ*^−1^, an energetically well-separated Kagome moiré band structure emerges at sufficiently small twist angles [**a**, right column, marked in blue]. **c** Spin–orbit interactions lift the quadratic touching of Kagome bands at Γ. The resulting band structure is gapped and realizes a novel Kagome moiré flat-band quantum spin Hall insulator with spin Chern numbers ± 1 of the top and bottom Kagome bands. **d** depicts the ab initio band structure of twisted bilayers for three representative twist angles with [right column] and without [left column] spin-orbit coupling, with the top-most valence bands arising from the emergent Kagome lattice. Colored lines indicate the continuum model band structure, fitted to the top valence bands [see main text]. Additional deeper *p*_*z*_ orbital valence bands appear > 50 meV below the band edge and are not accounted for in the continuum theory, however are progressively separated energetically from the Kagome bands as the twist angle is reduced. Importantly, spin-orbit interactions split off a topological band with spin Chern number ${{{{{{{{\mathcal{C}}}}}}}}}_{s}=1$ [thick blue line], as discussed in the main text. **e** Matching charge density distributions from the pseudospin continuum theory and ab initio calculations confirm the emergent Kagome band structure.

Figure 2a depicts the structure of the resulting moiré bands without spin-orbit coupling, as a function of scalar *V* ≡ *V*_1_ and pseudospin ${V}^{\prime}\equiv {V}_{1}^{\prime}$ potentials. For ${V}^{\prime}=0$, the scalar potential *V* localizes the hole charge density on a honeycomb lattice of AB/BA regions [Fig. 2a, left column; Fig. 2b (I)], and an energetically well-separated set of honeycomb bands with Dirac points at **K**, ${{{{{{{\bf{K}}}}}}}}^{\prime}$ emerges at the top of the valence band. These retain a twofold orbital *p*_*x*_, *p*_*y*_ character, with the degeneracy of the bands weakly broken due to orbital anisotropy *η* ≠ 0. This directly mirrors the low-energy band structure of twisted bilayer graphene, however with the two-orbital structure resulting from the *p*_*x*_, *p*_*y*_ degeneracy of the constituent states at Γ as opposed to a valley degeneracy.

However, already the next lower in energy (fifth) moiré valence band reveals upon closer inspection a charge density distribution with a Kagome pattern [Fig. 2b, pattern (II)], localized in the “X” regions of the moiré unit cell [Fig. 1e]. These states gain energy from a finite pseudospin potential ${V}^{\prime}$, which lifts them to higher energies: Beyond a critical ${V}^{\prime}$, the fifth “Kagome” band and the bottom *p*_*x*_, *p*_*y*_ honeycomb bands invert their energetic ordering at Γ. Consequently, the charge density distribution of the top *p*_*x*_, *p*_*y*_ bands shifts from AB/BA honeycomb regions to “X” Kagome sites [Fig. 2b, pattern (III)]. If the moiré potentials are sufficiently weak, the three resulting bands that constitute the emergent moiré Kagome lattice couple to a fourth moiré orbital centered on the hexagons of the lattice, with a charge density distribution that forms a ring around the AA regions of the moiré unit cell [Fig. 2b, pattern (IV)]. As the twist angle is further reduced, an energetically well-separated set of three Kagome lattice bands emerges as the top-most set of moiré valence states [Fig. 2b, patterns (V), (VI)].

### Ab initio characterization

The above behavior closely matches the results from large-scale ab initio calculations of the twisted moiré supercell, depicted in Fig. 2d, left column, for three representative twist angles [see Supplementary Note 1]. As the angle is reduced, a set of bands with a Kagome charge distribution at Γ splits off progressively from deeper valence bands. For the larger twist angles ≥2.28^∘^ that are still within computational reach for density functional calculations, this energetic separation is not yet sufficient to completely separate the Kagome bands of chalcogen antibonding *p*_*x*_, *p*_*y*_ character from states with *p*_*z*_ or bonding *p*_*x*_, *p*_*y*_ character (<50 meV below the band edge), not included in the continuum theory. Nevertheless, the top-most Kagome bands of interest are already well-captured via the continuum model for the smallest twist angle [Fig. 2d, bottom-left] upon accounting only for the lowest harmonic of the moiré potential.

Crucially, the inclusion of spin-orbit coupling [Eq. (3)] now opens up a gap at the Kagome Dirac points and lifts the quadratic band touching degeneracy at Γ [Fig. 2c], reflected in ab initio simulations with spin-orbit interactions [Fig. 2d, right column]. As the top-most valence states originate from *p*_*x*_, *p*_*y*_ orbitals at small twist angles, spin-flip spin-orbit interactions are negligible and spin-*z* remains a good quantum number. Remarkably, this results in three almost dispersionless moiré bands that realize a novel Kagome topological quantum spin Hall insulator with spin Chern numbers ${{{{{{{{\mathcal{C}}}}}}}}}_{s}=\pm \!1$ for the first and third flat band [Fig. 2c, d]. In marked contrast to conventional topological materials, however, while superlattice interference quenches the kinetic energy scales, spin-orbit coupling *λ*_soc_ enters as a bare atomic scale and hence becomes the *dominant energy scale* that governs the low-energy physics of the moiré valence bands in ZrS_2_. This highly-tunable materials realization of an “ultra-strong” spin-orbit interaction regime in a moiré heterostructure constitutes a central result of this paper.

To model the emergent top-most flat topological moiré band in twisted ZrS_2_, we proceed with a fit of the pseudospin continuum theory [Eq. (1)] to the spin-orbit-coupled ab initio band structure for *θ* = 2.64^∘^ [Fig. 2d, middle-right panel]. As the minimal model of Eq. (1) does not account for bonding *p*_*x*_, *p*_*y*_, or *p*_*z*_ states, the third-highest ab initio valence band (−50 meV below the valence band edge) is composed primarily of bonding *p*_*x*_, *p*_*y*_, and *p*_*z*_ orbitals and is excluded from the fit. We note that this band separates energetically from the three Kagome moiré bands at lower twist angles. We obtain excellent agreement for the top two bands of *p*_*x*_, *p*_*y*_ antibonding character using *η* = 0.33, *m*^⋆^ = 0.27*m*_0_, *λ*_soc_ = 57 meV, *V*_1_ = 5.5 meV, ${V}_{1}^{\prime}=-9.3\;{{{{{{{\rm{meV}}}}}}}}$, *V*_2_ = 11.5 meV, ${V}_{2}^{\prime}=-5.1\;{{{{{{{\rm{meV}}}}}}}}$. Scaling with twist angle similarly matches the ab initio band structure at 2.45^∘^ [Fig. 2d, bottom-right panel]. As expected, the top-most band is topologically nontrivial with spin Chern number ${{{{{{{{\mathcal{C}}}}}}}}}_{s}=\pm \!1$. Figure 2e compares the corresponding charge density distributions at Γ for ab initio and continuum model calculations; both exhibit comparable Kagome patterns as well as a competing band at lower energies with a ring-shaped charge pattern around the *AA* region, which similarly becomes energetically separated from Kagome bands at lower twist angles [Fig. 2a].

### Tight-binding description of emergent moiré Kagome bands

**Fig. 3:**
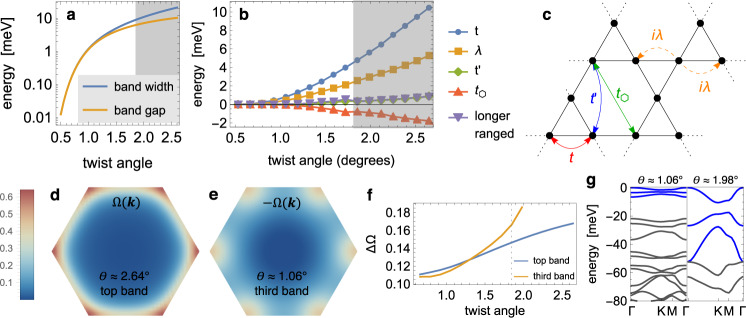
Twist angle dependence of the Kagome moiré lattice. **a** Band width and energetic separation of the top-most moiré valence band, extrapolated from the continuum theory as a function of twist angle. **b** Kagome tight-binding parameterization of the top three moiré bands, with real and imaginary hoppings depicted schematically in **c**. Shaded regions denote larger twist angles for which the third Kagome band does not remain well-isolated from lower-lying states; tight-binding parameterization in this region fits only the top two bands. **d** Berry curvature at 2.64^∘^ for the first moiré Kagome valence band, as well as **e** for the third band at 1.06^∘^. **f** Berry curvature fluctuations ΔΩ are suppressed as the twist angle is reduced, approaching a moiré realization of a Landau level. **g** Band structure of the continuum model at *θ* ≈ 1.06^∘^, 1.98^∘^.

A key advantage of the continuum theory is the possibility to study the behavior at small twist angles in a computationally feasible manner. Figure 3a depicts the bandwidth of the top-most topological moiré Kagome band, as well as the single-particle gap to the next deeper valence band, as a function of twist angle *a*_0_ ~ *θ*^−1^. The bandwidth of the top-most topological band decreases exponentially with twist angle, whereas the ratio between bandwidth and band gap saturates below ≈ 2^∘^ and approaches one. Below this twist angle, the three Kagome bands become fully isolated in energy from deeper valence states [Fig. 3g]. This immediately suggests a fruitful tight-binding parameterization at ultra-small angles, presuming that local lattice relaxation effects remain manageable. Results are shown in Fig. 3b for a tight-binding model depicted schematically in (c), but including up to 8th-neighbor hopping to ensure a good fit over all angles [see Supplementary Note 2]. For small angles ≪ 2^∘^, the top three bands become well-captured by a nearest-neighbor Kagome tight-binding model with imaginary hoppings. Third-neighbor hopping *t*_*δ*_ through the hexagons are leading corrections to this model and follow from the elliptical shapes of the charge density distribution at the Kagome “X” sites.

The sizable imaginary nearest-neighbor hopping [Fig. 3b] is a direct consequence of the strong spin–orbit coupling limit and can be interpreted as a finite effective staggered magnetic flux through the elementary triangles of the Kagome lattice. It lifts the quadratic touching of flat and dispersive Kagome bands and opens up a gap at the Dirac points, realizing a time-reversal-invariant version of a parent model for fractional Chern insulators^43,44^.

### Electronic interactions and spontaneous quantum anomalous Hall effect

The tunable realization of isolated time-reversal symmetric topological flat bands in twisted ZrS_2_ is an ideal starting point for the stabilization of a host of correlated topological states of matter, ranging from interaction-induced quantum anomalous Hall effects^45^ to elusive fractional Chern and topological insulators^46–49^. To investigate the role of electronic interactions and propensity for correlated topological phases in twisted ZrS_2_, we now study the top-most moiré Kagome band at fractional fillings and augment the effective three-band Kagome tight-binding description [Fig. 3]—derived from the continuum theory, and a continuous function of the twist angle—via a screened Coulomb repulsion. The interaction is constrained for simplicity to a local Hubbard ($U{\sum }_{i}{\hat{n}}_{i\uparrow }{\hat{n}}_{i\downarrow }$) and nearest-neighbor density ($({U}^{\prime}/2){\sum }_{ < ij > \sigma {\sigma }^{\prime}}\hat{n}_{i\sigma} \hat{n}_{j{\sigma }^{\prime}}$) interaction, expected to be a good approximation for screening due to metallic gates^50,51^. Suppose first that the top-most topological moiré Kagome band is tuned to half filling via electrostatic gating. A nontrivial spin Chern number precludes a straightforward Wannier tight-binding representation of this individual band. Instead, as deeper fully-filled valence bands are energetically separated, the low-energy behavior can be captured starting Kagome tight-binding model via projecting Coulomb interactions $U,{U}^{\prime}$ onto the Bloch states of a single fractionally-filled flat topological band, in direct analogy to lowest Landau level projections for the fractional quantum Hall effect. The resulting interacting problem is governed by an effective Hamiltonian

5 $${\hat{H}}_{{{{{{{{\rm{eff}}}}}}}}}=\mathop{\sum}\limits_{{{{{{{{\bf{k}}}}}}}}\sigma }{\epsilon }_{{{{{{{{\bf{k}}}}}}}}}{\hat{c}}_{{{{{{{{\bf{k}}}}}}}}\sigma }^{{{{\dagger}}} }{\hat{c}}_{{{{{{{{\bf{k}}}}}}}}\sigma }+\frac{1}{L}\mathop{\sum}\limits_{{{{{{{{{\bf{k}}}}}}}}{{{{{{{{\bf{k}}}}}}}}}^{\prime}{{{{{{{\bf{q}}}}}}}}}\atop {\sigma {\sigma }^{\prime}}}{V}_{{{{{{{{\bf{k}}}}}}}}{{{{{{{{\bf{k}}}}}}}}}^{\prime}{{{{{{{\bf{q}}}}}}}}}^{\sigma {\sigma }^{\prime}}{\hat{c}}_{{{{{{{{\bf{k}}}}}}}},\sigma }{\hat{c}}_{{{{{{{{{\bf{k}}}}}}}}}^{\prime},{\sigma }^{\prime}}{\hat{c}}_{{{{{{{{{\bf{k}}}}}}}}}^{\prime}-{{{{{{{\bf{q}}}}}}}},{\sigma }^{\prime}}^{{{{\dagger}}} }{\hat{c}}_{{{{{{{{\bf{k}}}}}}}}+{{{{{{{\bf{q}}}}}}}},\sigma }^{{{{\dagger}}} }$$

where ${\hat{c}}_{{{{{{{{\bf{k}}}}}}}}\sigma }^{{{{\dagger}}} },\hat{c}_{{{{{{{{\bf{k}}}}}}}}\sigma}$ create/annihilate electrons in the flat band with Bloch momenta **k**, *ϵ*_**k**_ denotes the residual band dispersion, *L* is the system size, and

6 $${V}_{{{{{{{{\bf{k}}}}}}}}{{{{{{{{\bf{k}}}}}}}}}^{\prime}{{{{{{{\bf{q}}}}}}}}}^{\sigma {\sigma }^{\prime}}=\frac{1}{2}\mathop{\sum}\limits_{\alpha {\alpha }^{\prime}}{v}_{\alpha {\alpha }^{\prime}}({{{{{{{\bf{q}}}}}}}})\,{u}_{{{{{{{{\bf{k}}}}}}}}}^{(\alpha \sigma )}{u}_{{{{{{{{{\bf{k}}}}}}}}}^{\prime}}^{({\alpha }^{\prime}{\sigma }^{\prime})}{\left[{u}_{{{{{{{{{\bf{k}}}}}}}}}^{\prime}-{{{{{{{\bf{q}}}}}}}}}^{({\alpha }^{\prime}{\sigma }^{\prime})}{u}_{{{{{{{{\bf{k}}}}}}}}+{{{{{{{\bf{q}}}}}}}}}^{(\alpha \sigma )}\right]}^{\star }$$

is the Coulomb repulsion projected to the Bloch states ${u}_{{{{{{{{\bf{k}}}}}}}}}^{(\alpha \sigma )}$ of the top-most band, derived from the tight-binding model, with

7 $$v({{{{{{{\bf{q}}}}}}}})=\left[\begin{array}{lll}U&{U}^{\prime}\cos \left(\frac{{{{{{{{\bf{k}}}}}}}}{{{{{{{{\bf{a}}}}}}}}}_{1}}{2}\right)&{U}^{\prime}\cos \left(\frac{{{{{{{{\bf{k}}}}}}}}{{{{{{{{\bf{a}}}}}}}}}_{2}}{2}\right)\\ {U}^{\prime}\cos \left(\frac{{{{{{{{\bf{k}}}}}}}}{{{{{{{{\bf{a}}}}}}}}}_{1}}{2}\right)&U&{U}^{\prime}\cos \left(\frac{{{{{{{{\bf{k}}}}}}}}{{{{{{{{\bf{a}}}}}}}}}_{3}}{2}\right)\\ {U}^{\prime}\cos \left(\frac{{{{{{{{\bf{k}}}}}}}}{{{{{{{{\bf{a}}}}}}}}}_{2}}{2}\right)&{U}^{\prime}\cos \left(\frac{{{{{{{{\bf{k}}}}}}}}{{{{{{{{\bf{a}}}}}}}}}_{3}}{2}\right)&U\end{array}\right]$$

**Fig. 4:**
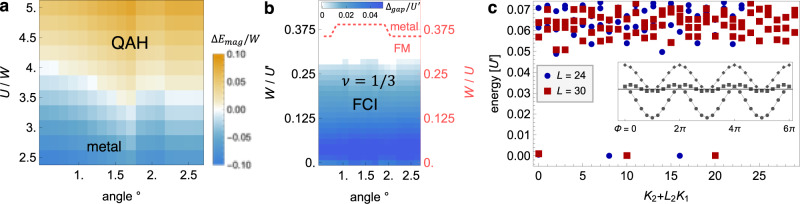
Quantum anomalous Hall effect and fractional Chern insulators. **a** At half filling, the first moiré band displays a ferromagnetic instability for a local Hubbard repulsion *U* on the order of ~4*W* the electronic bandwidth, depicted via the energy density gain from spontaneous spin polarization. Spin-polarized moiré quantum spin Hall bands realize a correlated quantum anomalous Hall phase. **b** At 1/6 hole doping, ferromagnetism (FM) persists for finite *U* [right axis] over a wide range of twist angles. Finite longer-ranged Coulomb repulsion ${U}^{\prime}$ [left axis] between nearest-neighbor Kagome sites drives this spin-polarized metal into a *ν* = 1/3 Laughlin phase, realizing a robust fractional Chern insulator, depicted in terms of the many-body excitation gap. **c** Fingerprint of the fractional Chern insulator in the low-energy spectrum for *L* = 24 (*L* = 30) unit cells with periodic boundary conditions and *N* = 8 (*N* = 10) fermions, depicted as a function of linearized total momentum (*K*_1_, *K*_2_). Inset depicts the characteristic spectral flow under insertion of a magnetic flux through the torus.

Here, momenta ${{{{{{{\bf{k}}}}}}}},{{{{{{{{\bf{k}}}}}}}}}^{\prime},{{{{{{{\bf{q}}}}}}}}$ are defined in the moiré Brillouin zone, **a**_*i*_ denote the moiré lattice vectors, and *α*, *σ* denote the sublattice and spin degrees of freedom. Since a sufficiently short-ranged interaction $U \; > \;{U}^{\prime}$ mainly imparts a local energetic penalty for electron pairs of opposite spin occupying the same Kagome “X” sites, a flat-band ferromagnetic instability generically ensues^52^ at half filling of the top-most quantum spin Hall band, in direct analogy to quantum Hall ferromagnetism^53^. The resulting spontaneous spin-polarized state is gapped and aligned in the *z* direction—it exhibits a quantum anomalous Hall effect by virtue of filling a quantum spin Hall band for one spin component only; this fully spin-polarized state is an exact gapped zero-energy ground state in the absence of dispersion^45^ and entails a quantized Hall conductivity. To study its robustness to the finite residual dispersion of the moiré band, we evaluate the phase diagram via exact diagonalization of Eq. (5) on a 4 × 4 unit cell cluster. Figure 4a depicts the phase diagram as a function of twist angle (which parameterizes the low-energy electronic band structure and Bloch states) and interaction strength *U* vs bandwidth *W* of the top-most band. A robust quantum anomalous Hall state emerges for interactions on the order of four times the moiré bandwidth and remains robust over a wide range of twist angles. Notably, the underlying mechanism is distinct from the observed quantum anomalous Hall effect in twisted bilayer graphene, relying instead on the *intrinsic* topologically non-trivial moiré band structure due to strong spin-orbit coupling and obviating the necessity for concurrent valley polarization and substrate effects.

### Fractional Chern insulators at fractional fillings

Having established a readily-accessible interaction-induced quantum anomalous Hall state at half filling, we now turn to the possibility of more exotic topologically-ordered phases at fractional fillings. Here, previous theoretical works established numerous examples of topological tight-binding models^46,54–57^ that a fractionally filled flat band with non-zero Chern number can in principle behave analogous to a fractionally filled Landau level and realize Abelian and non-Abelian fractional quantum Hall states in the absence of an external magnetic field. Uniformity of the Berry curvature is a key figure of merit^58^ and determines, jointly with the ideal droplet condition for the Fubini-Study metric^59–61^, the propensity for flat Chern bands to host fractional quantum Hall phases. Figure 3f quantifies Berry curvature fluctuations ${({{\Delta }}\Omega )}^{2}={\int}_{{{{{{{{\rm{BZ}}}}}}}}}{[{{\Omega }}({{{{{{{\bf{k}}}}}}}})-\sqrt{3}{{{{{{{\mathcal{C}}}}}}}}/8{\pi }^{2}]}^{2}d{{{{{{{\bf{k}}}}}}}}$ for the Kagome moiré bands, where ${{{{{{{\mathcal{C}}}}}}}}=\pm \!1$ is the spin Chern number. One finds that the Berry curvature flattens monotonically as the twist angle is reduced, with fluctuations substantially suppressed for the third Kagome valence band at small angles.

To demonstrate twisted ZrS_2_ as a candidate to observe FQH physics with external magnetic fields, we focus on the conceptually simplest Laughlin *ν* = 1/3 state at 1/6 hole doping, and study the interacting problem at small twist angles in exact diagonalization. Analogous to the half-filled case, electrons in the almost-flat band can avoid local Coulomb repulsion *U* via spontaneous spin polarization, yielding a robust ferromagnetic instability as a function of *U* [Fig. 4b, right axis, dashed line] over all investigated twist angles. However, spontaneous spin polarization due to *U* now leaves a single Chern band at 1/3 hole doping, with the resulting electronic phase governed by longer-ranged Coulomb interactions ${U}^{\prime}$. To study the propensity to realize a Laughlin state, we numerically investigate the resulting phase diagram as function of bandwidth $W/{U}^{\prime}$ [Fig. 4b, left axis]. For *W* = 0, corresponding to the Landau level limit of a perfectly-flat Chern band, our exact diagonalization calculations for 6 × 5 unit cells reveal a three-fold ground state degeneracy for periodic boundary conditions [Fig. 4c] with a gap to well-separated many-body excitations which persists as a function of system size. These ground states lie in three total momentum sectors that match the generalized Pauli principle for FCIs^56^, flow into each other upon adiabatic insertion of a magnetic flux through handles of the torus (periodic boundary conditions) and remain energetically separated from excitations, confirming the *ν* = 1/3 FCI^44,56^. Combined, these results indicate the robust stabilization of a fractional Chern insulator. The conclusions remain largely independent of the twist angle, and the FCI persists upon inclusion of finite band dispersion *W* until the many-body excitation gap closes for $W/{U}^{\prime} \sim 0.3$ [Fig. 4b, false color].

## Discussion

Having established a robust correlated quantum anomalous Hall phase at half filling and evidence for a *ν* = 1/3 fractional Chern insulator at one-sixth hole doping, an interesting follow-up question concerns the role of proximal deeper moiré valence bands, beyond the single-band approximation. For interactions that exceed the single-particle gap to other bands but remain smaller than the *overall* bandwidth of the three Kagome bands, the robustness of fractional Chern insulator phases has been well-documented^62^, in direct analogy to Landau level mixing in the conventional quantum Hall effect. A more substantial challenge however stems from details of possible longer-ranged electron interactions and exchange processes, which could serve to either enhance or suppress the stability of the fractionalized phases at different filling fractions. These processes sensitively depend on the screening environment and gating^50^, and microscopic calculations present a substantial methodological obstacle for twisted materials^63,64^. Conversely, analyzing the potential stability of more exotic yet more fragile non-Abelian quantum Hall states remains an interesting topic for future investigation. Furthermore, for sufficiently small twist angles, if the Coulomb repulsion exceeds the overall bandwidth of the three Kagome bands, sufficient screening could serve to form a local moment at overall half filling *ν* = 3/2. Such a Kagome Mott insulator would constitute a Moiré realization of a paradigmatic frustrated magnetic model, which has been under intense scrutiny for the potential to host an elusive quantum spin liquid phase.

Beyond the (fractional) quantum anomalous Hall effect, the realization of flat-band quantum spin Hall insulators further opens up the possibility to realize a myriad of unconventional ordered states of matter with non-trivial topology, including time-reversal invariant fractionalized phases, or topological superconductors. Consequently, twisted ZrS_2_ bilayers constitute a promising and tunable materials platform for such investigations, granting access to a novel and exotic regime of ultra-strong spin–orbit coupling that is not readily realizable in conventional crystalline solid-state systems. More broadly, a natural question concerns the extension of similar ideas of pseudospin potential engineering and strong spin-orbit coupling to other transition-metal dichalcogenide heterostructures such as TiS_2_ and HfS_2_ with  a multi-component character of the valence band edge. At the same time, the emergence of a moiré Kagome lattice from the fortuitous but robust interplay of geometry and interlayer coupling at small twist angles opens up a new pathway towards a moiré realization of magnetic phases in a paradigmatic frustrated system.

## Methods

### First-principles calculations

Ab initio calculations are performed with the Vienna Ab initio Simulation Package (VASP)^65^ based on density functional theory (DFT). Plane-wave basis sets are employed with an energy cutoff of 450 eV. The pseudopotentials are constructed with the projector augmented wave method^66^ and the exchange-correlation functionals are treated within the generalized gradient approximation (GGA)^67^. Only the Γ point is considered in the calculations due to the large size of the moiré supercells. A vacuum region larger than 15 Angstrom along the z-axis is applied to eliminate artificial interactions between periodic slab images. All atoms are relaxed until the forces on each atom are less than 0.01 eV/Angstrom. Van der Waals corrections are applied with the Tkatchenko-Scheffler method^68^ during the relaxation. The figures for the atomic structures and the charge density distributions are generated with the VESTA code^69^.

In this work, DFT calculations are only used to provide a reliable single-particle description of the top moiré valence bands of twisted bilayer ZrS_2_. The role of strong electronic interactions *within* the flat band is subsequently investigated in detail starting from the continuum theory and the effective tight-binding models described in the main text (with parameters extracted from the DFT calculations), using large-scale many-body exact diagonalization calculations (see below). Although the band gaps in the systems to the conduction band are underestimated by the DFT calculations at the GGA level, the conduction band lies at high energies and remains empty, hence does not affect the emergent many-body state at small hole doping of the flat moiré valence bands. Only the band dispersion and the shape of the moiré bands are relevant in this work and these are well captured by GGA. As shown in refs. ^38,70^, many-body corrections to GGA for 2D transitional metal dichalcogenides mainly appear as a rigid shift of the bands such that band gap is enlarged.

### Exact-diagonalization calculations

Fractional Chern insulating phases and the spontaneous quantum anomalous Hall effect are studied using exact diagonalization calculations of the many-body ground state of the projected interaction Hamiltonian [Eq. (5)]. Calculations are performed for *L*_1_ × *L*_2_ unit cell clusters with periodic boundary conditions and discrete momenta **k** = *n*_1_**b**_1_/*L*_1_ + *n*_2_**b**_2_/*L*_2_, with *n*_*i*_ = 0, …, *L*_*i*_ − 1 and reciprocal lattice vectors ${{{{{{{{\bf{b}}}}}}}}}_{1,2}={[2\pi ,\pm 2\pi /\sqrt{3}]}^{\top }$. Electron spin is explicitly included. Simulations of the interaction-induced quantum anomalous Hall effect at half filling are performed for 4 × 4 clusters. Results for *ν* = 1/3 fractional Chern insulators at 1/6 hole doping are obtained for 24-site (6 × 4) and 30-site (6 × 5) clusters.

## Supplementary information

Supplementary Information

## Data Availability

The raw data sets used for the presented analysis within the current study are available from the corresponding authors on reasonable request.

## Code availability

Custom codes used in this work can be provided by the corresponding author on reasonable request. Ab initio calculations were performed with VASP (version 5.4.4).
