# Supplementary material for: Ultra-strong spin–orbit coupling and topological moiré engineering in twisted ZrS2 bilayers
Source: Nat Commun. 2022 Aug 22;13:4915. doi: 10.1038/s41467-022-31604-w (PMC9395362; doi:10.1038/s41467-022-31604-w)
Supplement: Supplementary file 1 — Supplementary Information [file 41467_2022_31604_MOESM1_ESM.pdf]

Supplementary Information – Ultra-Strong Spin-Orbit Coupling and Topological Moiré Engineering in Twisted ZrS_2 Bilayers

(Dated: May 7, 2022)

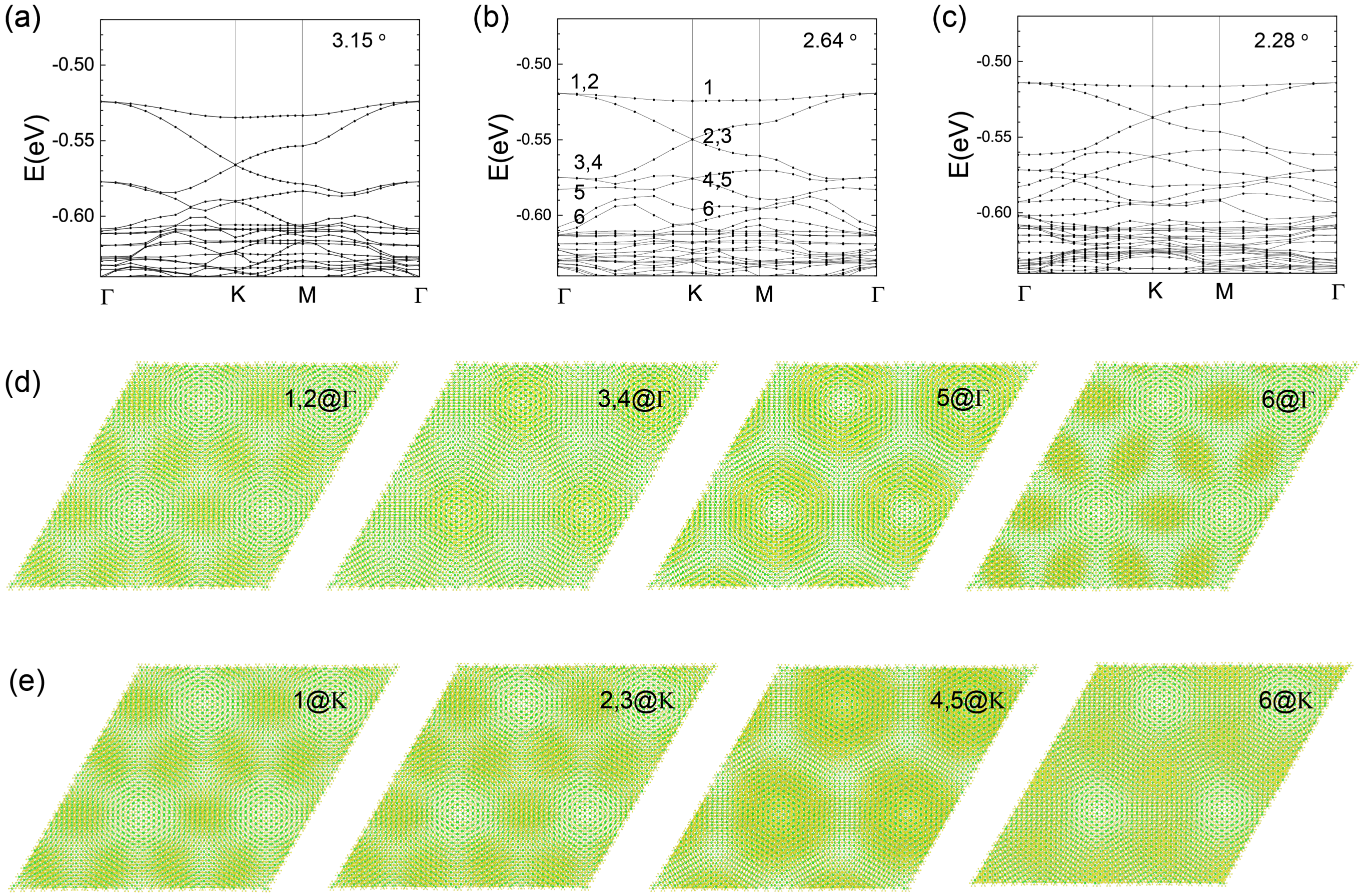

Supplementary Figure 1. **Electronic structures of twisted bilayers ZrS_2 calculated without spin-orbit coupling.** (a-c) DFT band structures of twisted bilayer ZrS_2 at twist angles 3,15, 2.64, and 2.28 degrees, respectively. (d-e)Charge density distributions of the top valence band states at the Gamma (d) and the K points (e) of twisted bilayer ZrS_2 at 2.64 degrees.

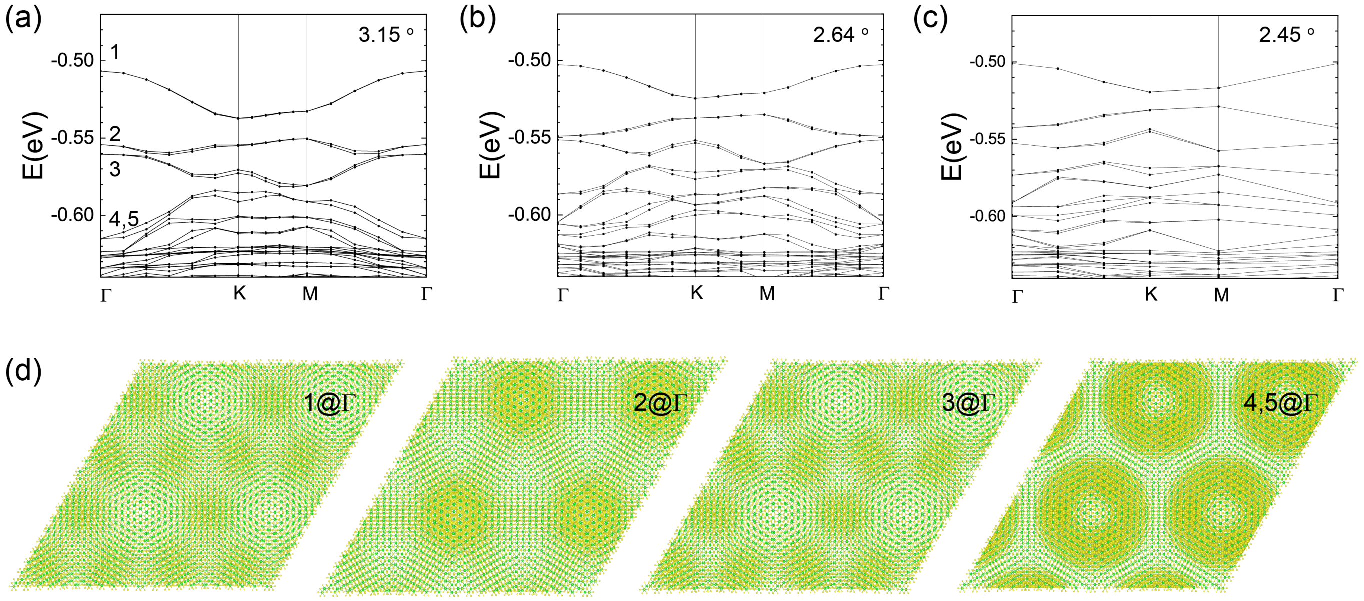

Supplementary Figure 2. **Electronic structures of twisted bilayer ZrS_2 calculated with spin-orbit coupling.** (a-c) DFT band structures of twisted bilayer ZrS_2 at twist angles 3,15, 2.64, and 2.45 degrees, respectively. (d)Charge density distributions of the top valence band states at the Gamma point of twisted bilayer ZrS_2 at 3.15 degrees.

SUPPLEMENTARY NOTE 1: ADDITIONAL ANALYSIS OF THE DFT BAND STRUCTURES

As shown in Supplementary Figure 1 for the calculations without spin-orbit coupling, the top valence bands of twisted bilayer ZrS_2 can be well fitted by the simple effective kagome tight-binding model discussed in the main text. For large twist angles, the lower half of the effective kagome bands are mixed and entangling with higher energy bands. The features matching well with the kagome lattice model become more obvious at smaller twist angles when the total band width decrease. At the smallest twist angle 2.28 degree that we can calculated with DFT (Supplementary Fig. 1(c)) , even the lower half of the effective kagome bands can be well recognized, albeit there are still some anti-crossing with the higher energy bands. To further confirm the characters of these bands, we extract the charge density distribution of the states at those bands as shown in Supplementary Fig. 1(e) and (f) for the case at 2.64 degrees. The charge density distribution of states at bands 1,2 and 6 at the Gamma point and bands 1,2,3 at the K point show a kagome lattice pattern, which is consistent with our expectation and further validate our tight-binding fitting. As shown in Supplementary Fig. Fig. 1(d), the total bandwidth W of the kagome bands decreases with twist angles while the energy separations between the top kagome band and the other bands at higher energy saturates as twist angle is smaller than 3 degrees. Therefore, when the twist angle further decreases (which is beyond our DFT calculations), the three kagome bands are expected to detach from the other bands as discussed in the main text.

When spin-orbit coupling is included, as shown in Supplementary Fig. 2 (and Fig. 1(f) in the main text), the top valence bands of twisted bilayer ZrS_2 are also well fitted to a effective kagome lattice tight-binding model discussed in the main text. By analyzing the band characters for the system at 3.15 degree as a typical example in Supplementary Fig. 2(e), we further confirm that the charge density distribution of the states at bands 1 and 3 at the Gamma point shows a kagome lattice pattern. As shown in Supplementary Fig. 2(d), similar to the results without spin-orbit coupling, when the twist angle decreases, the separations between the kagome bands and the other non-kagome bands saturates at around 50 meV while the total band width of the three kagome bands decreases. It is thus expected the three kagome bands can be eventually detached from the other bands at small twist angles.

SUPPLEMENTARY NOTE 2: EXPLICIT FORM OF THE EFFECTIVE LONG-RANGED KAGOMÉ TIGHT-BINDING MODEL

The main text details the construction of an effective tight-binding description from the strongly spin-orbit coupled continuum model. To accurately capture the top-most bands of Kagomé character, we employ a model with up to eigth-neighbor hoppings. For small twist angles, longer-ranged hoppings remain negligible. As discussed in the main text, the three Kagomé bands cease to remain spectrally isolated from deeper moiré valence bands for angles in excess of $\sim 1.65°$. As the gap to deeper bands closes, the fit proceeds with only the top two isolated Kagomé bands. Correspondingly, the range of (exponentially localized) hoppings increases to retain a minimal Kagomé tight-binding description. For completeness, the effective tight-binding model reads explicitly:

$$\hat{H} = \sum_{n=1}^{8} \hat{H}_n \tag{1}$$

where

$$\hat{H}_1 = t \begin{pmatrix} 0 & 2\cos\left(\frac{k_1}{2}\right) & 2\cos\left(\frac{k_2}{2}\right) \\ 2\cos\left(\frac{k_1}{2}\right) & 0 & 2\cos\left(\frac{k_1-k_2}{2}\right) \\ 2\cos\left(\frac{k_2}{2}\right) & 2\cos\left(\frac{k_1-k_2}{2}\right) & 0 \end{pmatrix} + \lambda \begin{pmatrix} 0 & 2i\cos\left(\frac{k_1}{2}\right) & -2i\cos\left(\frac{k_2}{2}\right) \\ -2i\cos\left(\frac{k_1}{2}\right) & 0 & 2i\cos\left(\frac{k_1-k_2}{2}\right) \\ 2i\cos\left(\frac{k_2}{2}\right) & -2i\cos\left(\frac{k_1-k_2}{2}\right) & 0 \end{pmatrix} \tag{2}$$

$$\begin{aligned} \hat{H}_2 = t' &\begin{pmatrix} 0 & 2\cos\left(\frac{k_1-2k_2}{2}\right) & 2\cos\left(k_1-\frac{k_2}{2}\right) \\ 2\cos\left(\frac{k_1-2k_2}{2}\right) & 0 & 2\cos\left(\frac{k_1+k_2}{2}\right) \\ 2\cos\left(k_1-\frac{k_2}{2}\right) & 2\cos\left(\frac{k_1+k_2}{2}\right) & 0 \end{pmatrix} \\ + \lambda' &\begin{pmatrix} 0 & 2i\cos\left(\frac{k_1-2k_2}{2}\right) & -2i\cos\left(k_1-\frac{k_2}{2}\right) \\ -2i\cos\left(\frac{k_1-2k_2}{2}\right) & 0 & 2i\cos\left(\frac{k_1+k_2}{2}\right) \\ 2i\cos\left(k_1-\frac{k_2}{2}\right) & -2i\cos\left(\frac{k_1+k_2}{2}\right) & 0 \end{pmatrix} \end{aligned} \tag{3}$$

$$\hat{H}_3 = t_1'' \begin{pmatrix} 2\cos(k_1 - k_2) & 0 & 0 \\ 0 & 2\cos(k_2) & 0 \\ 0 & 0 & 2\cos(k_1) \end{pmatrix} + t_2'' \begin{pmatrix} 2(\cos(k_1) + \cos(k_2)) & 0 & 0 \\ 0 & 2(\cos(k_1 - k_2) + \cos(k_1)) & 0 \\ 0 & 0 & 2(\cos(k_1 - k_2) + \cos(k_2)) \end{pmatrix} \tag{4}$$

$$\hat{H}_4 = t'''' \begin{pmatrix} 0 & 4\cos(k_1)\cos\left(\frac{k_1-2k_2}{2}\right) & 4\cos(k_2)\cos\left(k_1 - \frac{k_2}{2}\right) \\ 4\cos(k_1)\cos\left(\frac{k_1-2k_2}{2}\right) & 0 & 2\left(\cos\left(\frac{k_1-3k_2}{2}\right) + \cos\left(\frac{3k_1-k_2}{2}\right)\right) \\ 4\cos(k_2)\cos\left(k_1 - \frac{k_2}{2}\right) & 2\left(\cos\left(\frac{k_1-3k_2}{2}\right) + \cos\left(\frac{3k_1-k_2}{2}\right)\right) & 0 \end{pmatrix} + \lambda'''' \begin{pmatrix} 0 & 4i\cos(k_1)\cos\left(\frac{k_1-2k_2}{2}\right) & -4i\cos(k_2)\cos\left(k_1 - \frac{k_2}{2}\right) \\ -4i\cos(k_1)\cos\left(\frac{k_1-2k_2}{2}\right) & 0 & 2i\left(\cos\left(\frac{k_1-3k_2}{2}\right) + \cos\left(\frac{3k_1-k_2}{2}\right)\right) \\ 4i\cos(k_2)\cos\left(k_1 - \frac{k_2}{2}\right) & -2i\left(\cos\left(\frac{k_1-3k_2}{2}\right) + \cos\left(\frac{3k_1-k_2}{2}\right)\right) & 0 \end{pmatrix} \tag{5}$$

$$\hat{H}_5 = t''''' \begin{pmatrix} 0 & 2\cos\left(\frac{3k_1}{2}\right) & 2\cos\left(\frac{3k_2}{2}\right) \\ 2\cos\left(\frac{3k_1}{2}\right) & 0 & 2\cos\left(\frac{3(k_1-k_2)}{2}\right) \\ 2\cos\left(\frac{3k_2}{2}\right) & 2\cos\left(\frac{3(k_1-k_2)}{2}\right) & 0 \end{pmatrix} + \lambda''''' \begin{pmatrix} 0 & 2i\cos\left(\frac{3k_1}{2}\right) & -2i\cos\left(\frac{3k_2}{2}\right) \\ -2i\cos\left(\frac{3k_1}{2}\right) & 0 & 2i\cos\left(\frac{3(k_1-k_2)}{2}\right) \\ 2i\cos\left(\frac{3k_2}{2}\right) & -2i\cos\left(\frac{3(k_1-k_2)}{2}\right) & 0 \end{pmatrix} \tag{6}$$

$$\hat{H}_6 = t_1'''''' \begin{pmatrix} 2\cos(k_1 + k_2) & 0 & 0 \\ 0 & 2\cos(2k_1 - k_2) & 0 \\ 0 & 0 & 2\cos(k_1 - 2k_2) \end{pmatrix} + t_2'''''' \begin{pmatrix} 2(\cos(k_1 - 2k_2) + \cos(2k_1 - k_2)) & 0 & 0 \\ 0 & 2(\cos(k_1 - 2k_2) + \cos(k_1 + k_2)) & 0 \\ 0 & 0 & 2(\cos(2k_1 - k_2) + \cos(k_1 + k_2)) \end{pmatrix} \tag{7}$$

$$\hat{H}_7 = t''''''' \begin{pmatrix} 0 & 4\cos\left(\frac{k_1}{2}\right)\cos(k_1 - 2k_2) & 4\cos\left(\frac{k_2}{2}\right)\cos(2k_1 - k_2) \\ 4\cos\left(\frac{k_1}{2}\right)\cos(k_1 - 2k_2) & 0 & 2\left(\cos\left(\frac{3k_1+k_2}{2}\right) + \cos\left(\frac{k_1+3k_2}{2}\right)\right) \\ 4\cos\left(\frac{k_2}{2}\right)\cos(2k_1 - k_2) & 2\left(\cos\left(\frac{3k_1+k_2}{2}\right) + \cos\left(\frac{k_1+3k_2}{2}\right)\right) & 0 \end{pmatrix} + \lambda''''''' \begin{pmatrix} 0 & 4i\cos\left(\frac{k_1}{2}\right)\cos(k_1 - 2k_2) & -4i\cos\left(\frac{k_2}{2}\right)\cos(2k_1 - k_2) \\ -4i\cos\left(\frac{k_1}{2}\right)\cos(k_1 - 2k_2) & 0 & 2i\left(\cos\left(\frac{3k_1+k_2}{2}\right) + \cos\left(\frac{k_1+3k_2}{2}\right)\right) \\ 4i\cos\left(\frac{k_2}{2}\right)\cos(2k_1 - k_2) & -2i\left(\cos\left(\frac{3k_1+k_2}{2}\right) + \cos\left(\frac{k_1+3k_2}{2}\right)\right) & 0 \end{pmatrix} \tag{8}$$

$$\hat{H}_8 = t_1'''''''' \begin{pmatrix} 2\cos(2(k_1 - k_2)) & 0 & 0 \\ 0 & 2\cos(2k_2) & 0 \\ 0 & 0 & 2\cos(2k_1) \end{pmatrix} + t_2'''''''' \begin{pmatrix} 2(\cos(2k_1) + \cos(2k_2)) & 0 & 0 \\ 0 & 4\cos(k_2)\cos(2k_1 - k_2) & 0 \\ 0 & 0 & 4\cos(k_1)\cos(k_1 - 2k_2) \end{pmatrix} \tag{9}$$

Here, $t, t', \ldots$ and $\lambda, \lambda', \ldots$ denote n-th neighbor hopping and spin-orbit coupling amplitudes, respectively.
